# *Candida utilis* and *Chlorella vulgaris* Counteract Intestinal Inflammation in Atlantic Salmon (*Salmo salar* L.)

**DOI:** 10.1371/journal.pone.0083213

**Published:** 2013-12-27

**Authors:** Fabian Grammes, Felipe Eduardo Reveco, Odd Helge Romarheim, Thor Landsverk, Liv Torunn Mydland, Margareth Øverland

**Affiliations:** 1 Aquaculture Protein Centre, CoE, Department of Animal and Aquacultural Sciences, Norwegian University of Life Sciences, Ås, Norway; 2 Department of Basic Sciences and Aquatic Medicine, Norwegian School of Veterinary Science, Oslo, Norway; Univeristy of California Riverside, United States of America

## Abstract

Intestinal inflammation, caused by impaired intestinal homeostasis, is a serious condition in both animals and humans. The use of conventional extracted soybean meal (SBM) in diets for Atlantic salmon and several other fish species is known to induce enteropathy in the distal intestine, a condition often referred to as SBM induced enteropathy (SBMIE). In the present study, we investigated the potential of different microbial ingredients to alleviate SBMIE in Atlantic salmon, as a model of feed-induced inflammation. The dietary treatments consisted of a negative control based on fish meal (FM), a positive control based on 20% SBM, and four experimental diets combining 20% SBM with either one of the three yeasts *Candida utilis* (CU), *Kluyveromyces marxianus* (KM), *Saccharomyces cerevisiae* (SC) or the microalgae *Chlorella vulgaris* (CV). Histopathological examination of the distal intestine showed that all fish fed the SC or SBM diets developed characteristic signs of SBMIE, while those fed the FM, CV or CU diets showed a healthy intestine. Fish fed the KM diet showed intermediate signs of SBMIE. Corroborating results were obtained when measuring the relative length of PCNA positive cells in the crypts of the distal intestine. Gene set enrichment analysis revealed decreased expression of amino acid, fat and drug metabolism pathways as well as increased expression of the pathways for NOD-like receptor signalling and chemokine signalling in both the SC and SBM groups while CV and CU were similar to FM and KM was intermediate. Gene expression of antimicrobial peptides was reduced in the groups showing SBMIE. The characterisation of microbial communities using PCR-DGGE showed a relative increased abundance of *Firmicutes* bacteria in fish fed the SC or SBM diets. Overall, our results show that both CU and CV were highly effective to counteract SBMIE, while KM had less effect and SC had no functional effects.

## Introduction

The increased use of plant ingredients in aquafeeds can lead to reduced feed intake, feed utilisation and compromise fish health and welfare due to their content of a wide range of antinutritional factors and antigens [1], [2]. Plant ingredients such as soybean meal (SBM) or peas are generally regarded as good protein sources. However, feeding either full-fat, de-fatted (extracted) SBM or pea protein concentrate at high levels to Atlantic salmon (*Salmo salar* L.) causes an inflammatory reaction in the distal part of the intestine (enteropathy) [3], [4]. A similar reaction to SBM can be found in other teleost species such as rainbow trout (*Oncorhynchus mykiss* W.) and common carp (*Cyprinus caprio* L.) [5], [6]. SBM induced enteropathy (SBMIE) starts at dietary inclusion levels of ca. 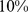 SBM and progresses in a dose dependent manner [7], [8]. The phenotype of the disease develops relatively rapid with the first histological signs of inflammation appearing after a couple of days and a fully developed SBMIE with almost 100% prevalence after three weeks of feeding SBM [8], [9]. Removing SBM from the diet of Atlantic salmon suffering SBMIE restores intestinal health [8]. Although the etiology of SBMIE is to date unknown, several causes for SBMIE have been proposed, and these include: antinutritional factors such as saponins [1], [10], unidentified antigens in SBM [11] as well as SBM induced changes of the intestinal microbiota [12]. Even though the causative agent for SBMIE has yet to be identified, it can be removed from SBM through alcohol extraction [13], [14].

It has been suggested that SBMIE in fish may resemble chronic intestinal inflammatory diseases in mammals such as coeliac disease (CD) or inflammatory bowel disease (IBD). CD is caused by a reaction to gluten [15] while IBD is thought to result from multiple factors such as loss of tolerance to the normal commensal microbiota, genetic susceptibility and environmental factors [16].

Our group recently showed that inclusion of a specific microbial ingredient (bacterial meal), obtained through fermentation of *Methylococcus capsulatus* in aquafeeds can prevent the development of SBMIE in Atlantic salmon [17]. *M.capsulatus* affects the development of SBMIE in a dose dependent manner, whereby dietary inclusion of approximately 15% *M.capsulatus* into the feed has been shown to be sufficient to prevent enteritis induced by 20% SBM [18]. Although, the data conclusively shows a functional feed effect *M.capsulatus*, the nature of this effect is currently unknown. Romarheim *et al.* recently showed that the large molecular fraction was responsible for the protective effects of *M.capsulatus*, suggesting that bacterial cell wall components could be the effectors [19].

Based on the success of *M.capsulatus* in preventing SBMIE, we hypothesised that other microbial ingredients may also have potential to counteract SBMIE in Atlantic salmon. In this paper, we report the functional effects of four different microbial ingredients in the SBMIE model in Atlantic salmon. The microbial ingredients include three different yeasts and one microalgae strain. The yeast strains are: *Saccharomyces cerevisiae* (SC), *Kluyveromyces marxianus* (KM) and *Candida utilis* (CU). KM and CU have recently been shown to be suitable protein sources for aquafeeds, while SC appeared to be less suitable [20]. The algae strain is *Chlorella vulgaris* (CV), a freshwater microalgae with a high nutrient content [21] and with a wide range of bioactive components [22], that have been used as a functional food. The effects of the different microbial ingredients were evaluated through the assessment of transcriptomic, histopathological immunomorphometric and bacterial changes in the distal intestine of Atlantic salmon. Our results show that substitution of FM by either CV or CU in aquafeed containing 20% SBM is effective to counteract SBMIE.

## Results

During the four week feeding experiment, the fish were fed either a fish meal (FM) diet (negative control), a diet containing SBM (positive control) or a diet containing SBM in combination with one of the four microbial ingredients (Table 1). At the end of the experiment, we found that the different feeding groups showed no difference with respect to gut, stomach or pyloric caeca weight (Table 2). No significant difference in weight gain was found, although this experiment was not designed to evaluate growth performance (see [20] for growth performance of CU KM and SC). The relative weight of the mid-intestinal section was significantly higher in the KM group compared to FM and the relative weight of the distal intestine was significantly lower in the SBM and SC groups compared to FM. No mortality was observed during the course of the experiment.

**Table 1:** Ingredients and chemical composition of the experimental diets.

	FM	SBM	CV 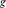	CU 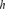	KM 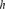	SC 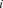
Ingredients [ 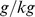 ], as is						
Fish meal 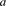	710	510	295	295	295	295
SBM 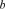	–	200	200	200	200	200
Microbial ingredient	–	–	200	200	200	200
Gelatin 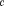	75	75	75	75	75	75
Potato starch 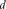	75	75	75	75	75	75
Fish oil 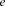	135	135	150	150	150	150
Min+Vit 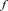	5	5	5	5	5	5
Analysed chemical composition [ 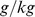 ], as is						
Dry matter	943	920	918	909	921	914
Crude protein	566	516	477	471	470	471
Crude lipid	192	177	173	168	175	168
Starch	120	113	139	112	134	112
Ash	103	85	66	64	70	64

FM: fish meal; SBM: soybean meal; CV: *Chlorella vulgaris*; CU: *Candida utilis*; KM: *Kluyveromyces marxianus*; SC: *Saccharomyces cerevisiae*.

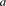
 Norse-LT 94, Nordsildmel, Egersund, Norway.

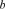
 Denofa, Fredrikstad, Norway.

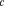
 Rousselot SAS, Courbevoie, France.

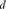
 Lyckeby Culinar AB, Fjlkinge, Sweden.

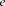
 NorSalmOil, Norsildmel, Egersund, Norway.

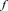
 Vitamin and mineral premix provided (per kg diet): all-trans retinyl acetate, 860 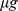; cholecalciferol, 37.5 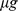; D,L-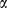-tocopherol acetate, 200 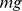; menadione, 10 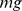; thiamin, 15 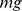; riboflavin, 25 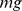; nicotinic acid, 75 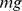; pantothenic acid, 30 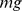; pyridoxine, 15 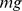; folic acid, 5 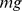; cyanocobalamin, 20 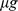; ascorbyl monophosphate, 125 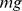; biotin, 0.25 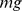; Ca, 1.1 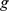; ZnSO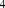, 296 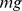; MnSO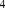, 41 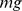; 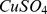, 13 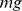; 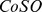, 2.6 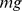; CaI, 3.5 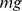; astaxanthin, 175 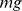.

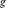
 Synergy Natural Products Pty Ltd, Sydney, Australia.

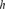
 Borregaard ASA, Sarpsborg, Norway.

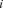
 YBD-Yeast Brewers, Sigma-Aldrich, St. Louis, USA.

**Table 2:** Fish weights and organ indexes.

	FM	SBM	CV	CU	KM	SC
Start weight [ 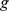 ]	107	107	107	107	106	107
Weight gain [ 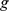 ]	42.4	38.5	31.2	33.5	34.1	28.7
Relative organ weights [%]						
Gut	7.62	7.78	7.43	7.76	7.8	7.77
Liver	1.37	1.23	1.44	1.48	1.41	1.42
Stomach	0.81	0.86	0.81	0.87	0.81	0.75
PC	3.67	3.62	3.56	3.48	3.66	3.69
Mi	0.21 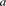	0.23 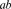	0.23 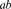	0.25 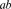	0.26 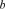	0.22 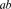
Di	0.57 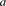	0.45 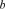	0.51 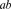	0.52 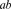	0.52 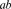	0.46 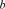

Relative organ weights are given in %, and calculated as 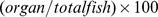. All rsults are shown as mean, statistical unit is the mean of the tank (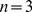; 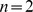 for BM). 20 fish were used to calculate the tank mean. Mean values with unlike superscript letters are significantly different (*p-value* 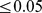; Tukey test). **PC**: pyloric caeca; **Mi**: mid intestine; **Di**: distal intestine.

### Intestinal histopathology

Assessing the histopathological changes in the distal intestine, we observed that fish from one tank of the SBM group showed uniform, but aberrant results from the other tanks of the SBM group. Because this observation was also inconsistent with results from previous experiments [17], [18], all samples originating from that tank were considered outliers and excluded from all subsequent analysis.

**Figure 1:**
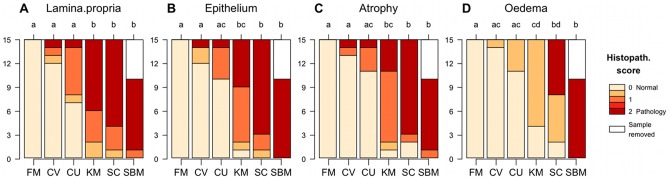
Histopathological changes in the distal intestine. The histopathology scores from the different feeding groups are plotted as stacked bars. Each stacked bar shows the histopathology scores of 15 fish (x-axis) that were sampled per diet. Histology scores were given for the four categories: **A**: *lamina propria*; **B**: Epithelium; **C**: Atrophy; **D**: Oedema. Groups with different letters on the upper x-axis are significantly different (multiple comparisons of mean ranks, Nemenyi-Dunn, 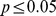).

All fish fed the SBM or SC diets developed characteristic signs of SBMIE, characterised by an accumulation of leukocytes in the *lamina propria*, a reduced vacuolization and cellular height of the epithelial cells, atrophy with shortening of the simple and complex intestinal folds and widening of the *lamina propria* and submucosa due to cellular infiltration and oedema (Fig. 1 and Fig. S1) [8]. Fish fed the KM diet showed somewhat less severe signs of oedema, but were statistically indistinguishable from both fish fed the SBM and SC diets with respect to the histological scores of *lamina propria*, epithelium and atrophy. Groups fed either the CV or CU diets showed only minor signs of SBMIE in the distal intestine, which were not statistically different from the FM group.

**Figure 2:**
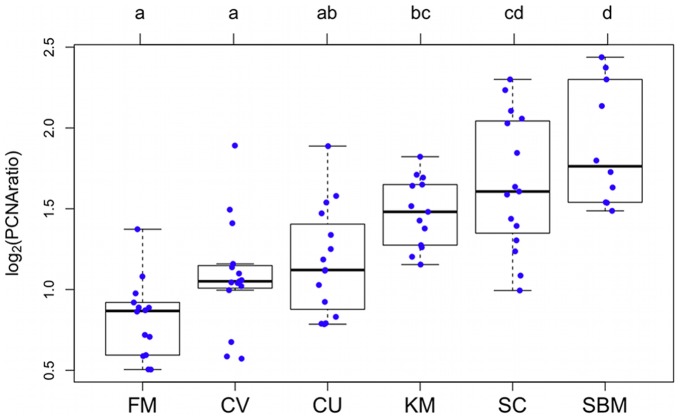
Morphometry of the distal intestine. Box-and-whiskerplots displaying the 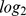 transformed relative length of the PCNA positive regions in the crypts of the distal intestine on the y-axis and the six feeding groups on the x-axis. Boxes mark the interquartile range with the black bar marking the median and whiskers extending to 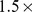 the interquartile range. Data points from the six different groups are plotted with a jitter in the x-direction for better visualisation. Groups with different letters on the upper x-axis are significantly different (multiple comparisons of means, Tukey, 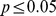).

Measurements of the stretches of PCNA positive cells in the crypts of distal-intestinal folds showed a gradual increase of the median length (FM 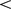 CV 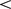 CU 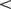 KM 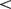 SC 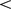 SBM, Fig. 2). Samples from both the SBM and SC groups showed the longest relative stretches of PCNA positive cells, whereas the FM, CV and CU groups showed the shortest, while the KM group was intermediate. The results from the immunomorphometrical analysis of the intestine supported the histopathology results, as indicated by high correlation (Table S1).

### Gene expression

A salmonid microarray was used to analyze gene expression in the distal intestine of 10 individual fish per group (seven from the SBM group), resulting in a total of 57 microarrays. For each of the five study groups (SBM, SC, KM, CV and CU) differentially expressed (DE) probes were identified by comparing gene expression levels of the study group to those of the negative control group (FM). Fold changes were calculated in *log* scale in a similar manner (see Materials and Methods).

**Figure 3:**
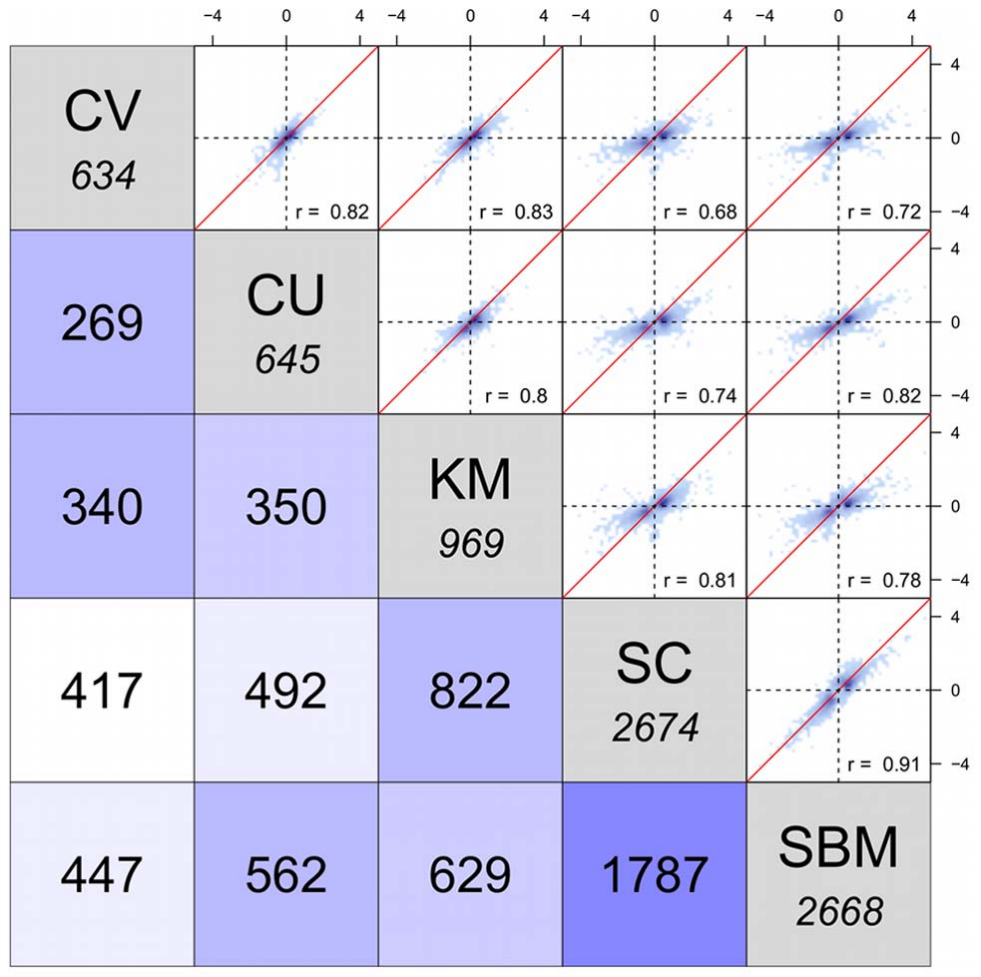
Correlation of differentially expressed genes. Gene expression changes in the distal intestine of the study groups: CV, CU, KM, SC and SBM, each relative to the negative control group (FM) were correlated against each other. The **upper triangle** shows the scatter plots and Pearson correlations (*r*) of the 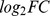 for 3,791 genes that showed DE in at least one of the study groups. The **diagonal** displays the total number of DE genes for each study group, while the number of genes that are commonly DE between two groups are shown in the **lower triangle**.

In total 3791 (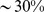 of the microarray) probes showed DE in the five study groups as a result of the different dietary treatments. For those 3791 probes, pair-wise correlation of the 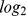 fold changes and overlap of the DE probes (Fig. 3) indicated a very high similarity of the transcriptional response in the SBM and SC groups (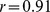). Both groups showed with 2668 and 2674 by far the highest numbers of DE probes and the strongest fold changes. 1787 probes were commonly DE in both groups. The remaining three groups (CV, CU and KM) formed a cluster based on strong correlation among each other (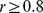), a feed-induced decrease in the scale of fold changes and distinct lower numbers of DE probes. Within the cluster, KM stood out for showing intermediate numbers of DE probes (969) and for showing strong correlation not only with the CV and CU groups, but also with the SBM and SC groups. Comparison of the KM and SC groups was particularly interesting, because both groups shared approximately 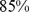 of the probes that were DE in the KM group. Both the CV and CU groups showed with 634 and 645 the lowest number of DE probes and are thus microbial ingredients with the strongest effect on alleviation of SBM-induced gene expression. The two groups generally showed low similarity with SC or SBM, respectively. It should be noted that simultaneous inclusion of SBM together with one of the microbial ingredient in none of the cases changed the overall direction of the expression, but it reduced the scale. A list of all DE probes can be found in Dataset S1.

**Figure 4:**
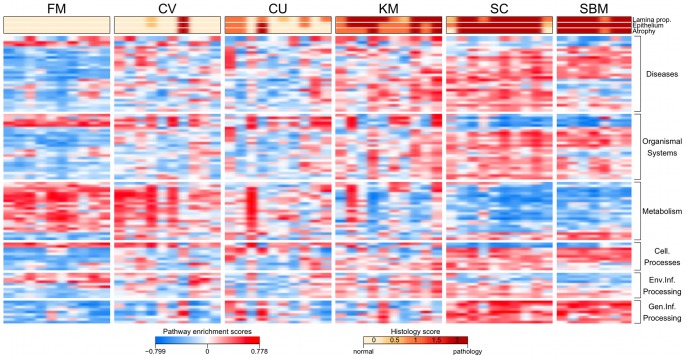
Dietary regulation of KEGG pathways. Heat map showing pathway enrichment scores for the 109 KEGG pathways that were significantly affected by the diet (ANOVA, adjusted *p-value* 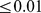). The pathways are grouped by function. Histology scores for *lamina propria*, epithelium and atrophy are colour coded and shown on top of the heat map.

To facilitate a **functional interpretation** of the high number of DE probes, gene set enrichment was assessed using Kyoto Encyclopedia of Genes and Genomes (KEGG) pathway annotations [23] to define gene sets (see Materials and Methods). The results of the gene set enrichment analysis showed that a total of 109 KEGG pathways were differentially regulated in the five study groups as a result of the different dietary treatments (Fig. 4). Additional tables containing all results from the gene set enrichment analysis can be found in the supplementary data (Dataset S2, S3). Overall, we observed that the pathway patterns were generally similar among the FM, CV and CU group, whereas the regulation of the pathways in the SC and SBM group was primarily opposite to that of the FM group. Pathway regulation in the KM group appeared to be in-between those of the FM and SBM group with individuals that received a lower pathology resembling the pathway pattern of the FM group and those with a higher pathology score resembled the SBM pattern. A more comprehensive description and interpretation of relevant KEGG pathways, grouped by function, is given in the following sections.

#### Genetic information processing

A set of nine pathways associated with genetic information processing showed the largest changes. All pathways showed a strong, significant up-regulation in both the SC and SBM group and only weak up-regulation in the remaining three groups. The set contains pathways related to translational processes (i.e. protein processing in endoplasmic reticulum or ribosome biogenesis in eukaryotes) as well as pathways that are involved in protein and RNA degradation (i.e. RNA degradation and Ubiquitin-mediated proteolysis). Taken together, these changes indicate a strong increase in protein turnover in both the SC and SBM groups.

#### Metabolism

The majority of the 23 metabolic affected pathways were related to amino acid and lipid metabolism, and showed a strong down-regulation in the groups SBM and SC, and to some extent in KM, while CV and CU were minimally affected. The two most significant down-regulated pathways were tryptophan metabolism and drug metabolism – cytochrome P450. Besides being a supplier of metabolic energy, tryptophan has two more highly important physiological functions in the gut. These are serving as a precursor for serotonin and regulating production of antimicrobial peptides. In the gut, serotonin has been shown to act as an immune regulator and to positively regulate bowel movements [24]. The gene *tryptophan 5-hydroxylase 1* (*THP1*), which was one of the two genes showing the strongest up-regulation, is responsible for catalysing the first step of serotonin synthesis.

**Figure 5:**
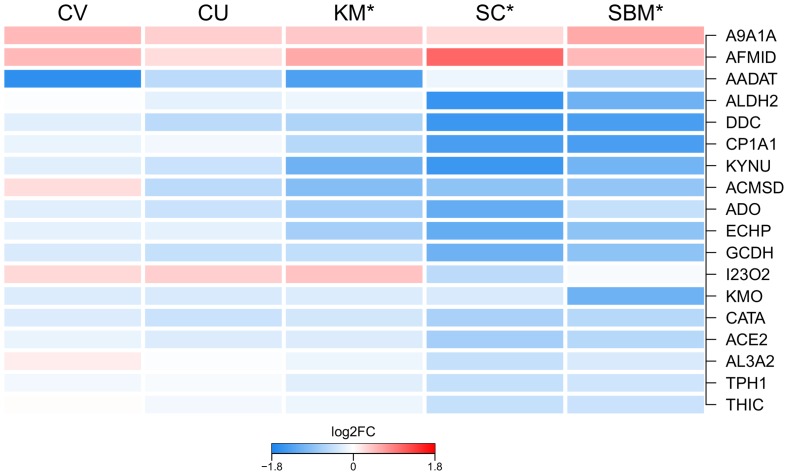
Regulation of tryptophan metabolism. Heat map showing the mean gene expression of the 18 genes in the KEGG pathway tryptophan metabolism, per group relative to the FM group. An asterisks(*) labelling the group indicates significant difference between the respective group and FM. *Aldehyde dehydrogenase family 9 member A1-A* (A9A1A); *Probable arylformamidase* (AFMID); *Kynurenine/alpha-aminoadipate aminotransferase* (AADAT); *Aldehyde dehydrogenase, mitochondrial* (ALDH2); *Aromatic-L-amino-acid decarboxylase* (DDC); *Cytochrome P450 1A1* (CP1A1); *Kynureninase* (KYNU); *2-amino-3-carboxymuconate-6-semialdehyde decarboxylase* (ACMSD); *Aldehyde oxidase* (ADO); *Peroxisomal bifunctional enzyme* (ECHP); *Glutaryl-CoA dehydrogenase* (GCDH); *Indoleamine 2,3-dioxygenase 2* (I23O2); *Kynurenine 3-monooxygenase* (KMO); *Catalase* (CATA); *Angiotensin-converting enzyme 2* (ACE2); *Fatty aldehyde dehydrogenase*(AL3A2); *Tryptophan 5-hydroxylase 1* (TPH1); *Acetyl-CoA acetyltransferase* (THIC).

In mice, metabolites of tryptophan generated via the tryptophan-nicotinamide pathway can regulate the production of antimicrobial peptides in the intestine [25]. Our study revealed that several genes involved in this process, such as *indolamine 2,3 dioxygenase* (*I23O2*), *kynurenine 3 monooxigenase* (*KMO*), *arylformamidase* (*AMFID*) and *kynurinase* (*KYNU*), were down-regulated. In addition, we found the gene *Angiotensin-converting enzyme 2* (*ACE2*) that is required for the expression of the neutral amino acid transporter *B* 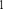 *AT1* on the luminal surface of epithelial cells [25], to be down-regulated. *ACE2* did not possess a corresponding KEGG pathway annotation, however, we found that its expression reflected the regulation of the tryptophan metabolism pathway (Fig. 5).

**Drug metabolism – cytochrome P450** was mainly characterised by down-regulation of several genes encoding Glutathion S transferases (GSTs) and UDP-glucuronosyltransferases (UGTs). Both UGTs and GSTs are phase II enzymes that detoxify electrophilic and cytotoxic substrates. Mucus-associated GST catalyzes the conjugation- detoxification of luminal reactive electrophiles, cytotoxic substrates and is thus able to prevent damage of the intestinal epithelium [26].

#### Organismal systems

This set comprised of 26 affected pathways showed mixed results with mostly up-regulation of the pathways in the SC and SBM groups but also some down-regulation. Among the pathways that showed the strongest down-regulation in both the SC and SBM groups was the pathway **bile secretion**. The genes of this pathway also include genes that regulate bile acid re-absorbtion through enterocytes. Important, down-regulated genes that belong to this pathway were the *Farnesoid X receptor* (*FXR* or *NR1H4*) and the bile salt transporters *ABCG5* and *S22A7*. FXR functions as a sensor for bile acid in the enterocytes, where its activation functions as a repressor for bile acid synthesis in the liver [27]; *ABCG5* and *ABCG8* together facilitate the efflux of bile acids from the enterocyte back into the intestinal lumen, their expression has shown to be regulated directly by dietary levels of cholesterol [28]. Bile acids are secreted into the intestine where they facilitate absorption of lipids, minerals, vitamins but also function as important signalling molecules. In Atlantic salmon, it has recently been shown that feeding SBM depletes the fish of cholesterol, probably through decreased intestinal bile re-absorption coupled with an increase in hepatic production [29]. In contrast to our study, Kortner *et al.* found higher expression of *FXR* in the distal intestine of SBM fed Atlantic salmon [29]. Nevertheless, a down-regulation in the pathway bile-secretion, likely related to decreased levels of total bile acids in the SC and SBM groups, matches down-regulation of the pathways **fat digestion and absorption**, **vitamin digestion and absorption** and **mineral absorption**.

Additionally, we found down regulation of three aquaporins *AQP1*, *AQP4* and *AQPA*, of which *AQP4* was the most significant. Aquaporins are water channels facilitating fluid transport across the epithelium. Transcriptional down-regulation of aquaporins can be observed in mammalian IBD models and in Atlantic salmon suffering SBMIE [10], [30].

With respect to immune-related pathways, we found among others **NOD-like receptor (NLR) signaling**, **chemokine signaling** and **leukocyte transendothelial migration** to be significantly up-regulated in both the SBM and SC groups. NLRs are pattern recognition receptors that can be found in epithelial cells of the intestine, where they can regulate the inflammatory response and production of antimicrobial peptides in response to microbial associated molecular patterns [31]. Polymorphism of the NLR NOD2 is a well-known risk factor for developing IBD [31], a condition that is found in humans and which shows similarities to SBMIE. Chemokine signalling is crucial for the recruitment of effector cells to the site of inflammation. The C-C motif chemokines 20 and 28 were among the highest induced genes in this pathway, both are important attractors in response to epithelial injury [32].

Several **nervous system** related pathways (axon guidance, glutamatergic synapse dopaminergic synapse, GABAergic synapse and GnRH signaling pathway) showed up-regulation in all the five different study groups. For all pathways, the up-regulation was strongest in the SBM and SC group and weakest in CV and CU. This could be indicative for structural and functional changes of the enteric nervous system, which can be observed in mammalian models for intestinal inflammation [33].

#### Diseases

Most of the 30 disease-related pathways were specifically related to human conditions like alcoholism and morphine addiction and were hence regarded as non informative for our experiment. Nevertheless, **pathogenic** ***Escherichia coli*** **infection** was the pathway showing the lowest adjusted *p-value* and up-regulation in all five study groups, whereby up-regulation in the SC and SBM groups was strongest followed by the KM group. The hallmark of enteropathogenic *E.coli* infections is the destruction of the intestinal microvillus brush border through restructuring of the underlying cytoskeleton, which is caused by signal transduction between bacterial and host cells. Thereby, causing diarrhoea in the host [34].

#### Environmental information processing

This set contained 10 affected pathways associated with envionmental information processing. Pathways of particular interest were: wnt signal processing, cell adhesion molecules and mTOR signalling. Activation of **wnt signalling** in the intestine is intrinsically related to holding the crypt-cells in a proliferative state [35]. Because this pathway proved to be up-regulated the most in both the SC and SBM groups, and a little less in the KM group, a functional relation to the increase in epithelial cell proliferation in the crypts of the distal intestinal folds (Fig. 2) appears coherent. **mTOR signalling** as the most significant pathway in this set was down-regulated in all study groups except in the KM group. The mTOR signalling pathway acts as a cellular nutrient sensor and regulates down-stream pathways like cell proliferation, protein synthesis and transcription [36]. Recently, it has been proposed that the mTOR pathway also acts as mediator of tryptophan mediated antimicrobial peptides expression [25].

Interestingly, we found a marked significant down-regulation of **cell adhesion molecules** in the SC and SBM groups. This included down-regulations of genes encoding Claudins (*CLD14, 15* and *18*) and *Cadherin 2* (*CADH2*). Claudins are a group of transmembrane proteins that participate in the formation of tight junctions and that regulate permeability [37]. CADH2 is a calcium dependent cell-cell adhesion glycoprotein. Interestingly, defects of the gene *Cadherin 1* (*CADH1*) are a known predisposing factor for IBD [38].

#### Cellular processes

A set of 11 affected pathways was related to cellular processes, of which the most interesting pathways were **endocytosis**, **lysozyme** and **peroxisome**. The former pathway was up-regulated in both SC and SBM while the latter were significantly down-regulated in all study groups.

#### Antimicrobial peptides

**Figure 6:**
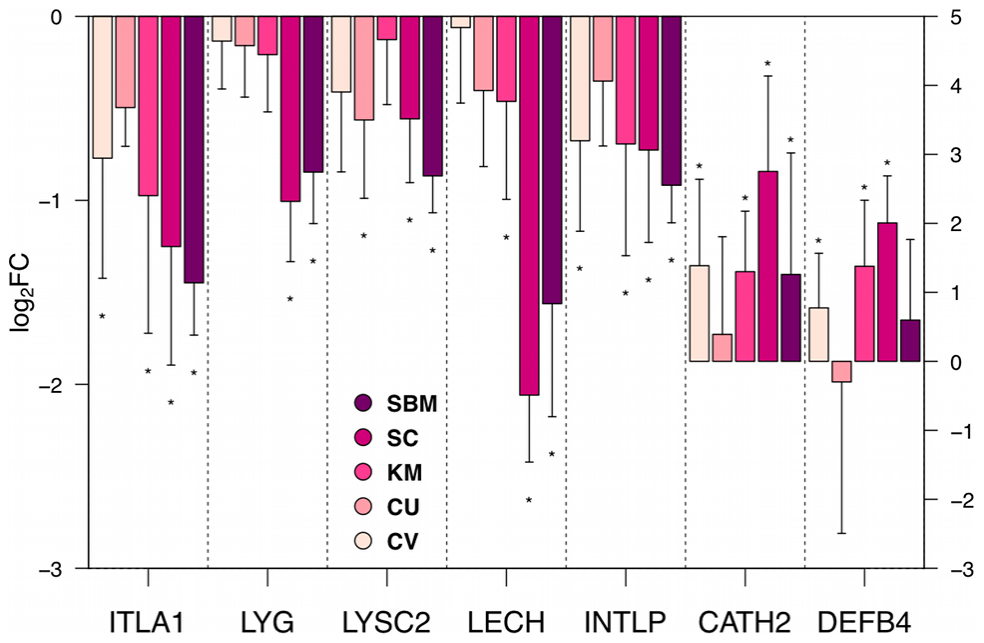
Expression of antimicrobial peptides. Barplots showing the gene expression as mean 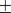 SD of log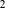 fold changes relative to FM. An asterisks(*) labelling the bar indicates significant diffrence (*p-value* 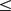
0.05) between the gene expression of the respective group and FM. Genes displayed are: *ITLA1* 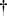: *Intelectin-1a*; *LYG* 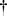: *Lysozyme G*; *LYSC2* 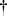 *Lysozyme C2 precursor*; *LECH* 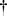: *Hepatic lectin*; *INTLP* 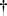: *Intelectin-like protein*; *CATH2* 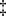: *Cathelicidin2*; *DEFB4* 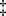: *Defensin B4*. The expression levels of the last two genes are displayed on the secondary y-axis. 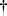: Gene represented on the microarray (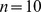). 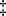: Gene measured by qRT-PCR (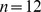).

Because we observed significant regulation of NLR signalling, tryptophan metabolism and mTOR signalling in the groups that showed stronger signs of SBMIE (SBM, SC and KM), we decided to investigate gene expression of antimicrobial peptides in the distal intestine. Intestinal secretion of antimicrobial peptides can protect the host against enteric bacterial pathogens and maintain homeostasis of the intestinal microbiota [39]. Six antimicrobial peptides were represented on the microarray, two were measured by qRT-PCR. Overall, most of the antimicrobial peptides showed stronger down-regulation (compared to FM) in the groups that were more affected by SBMIE (Fig. 6). Lysozymes (*LYG*, *LYSC2*) were significantly down-regulated in both the SBM and the SC groups. Expression of Lectins (*ITLA1*, *LECH* and *INTLP*) was significantly lower in the SBM, SC and KM groups. For *ITLA1* and *INTLP*, a significantly lower expression was also found for the CV group. In contrast, the expression of *Cathelicidin 2* (*CATH2*) and *Defensin B4* (*DEFB4*) was significant higher in the groups CV, KM and SC. *CATH2* also showed a significant higher expression in the SBM group.

### Microarray validation

**Figure 7:**
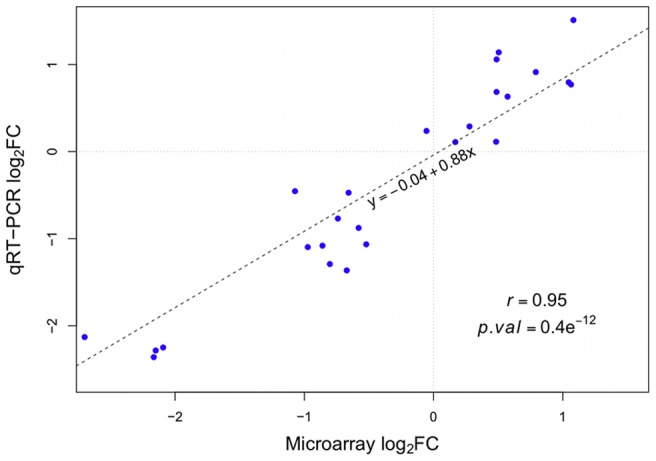
Validation of microarray results by qRT-PCR. Log
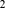
FCs from the microarray experiment are plotted on the *x-axis*, while log
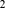
FCs from the qRT-PCR are plotted on the *y-axis*. qRT-PCR data was normalised to an index of the genes EF1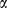 and GAPDH. Expression levels of five different genes were compared. For each gene the log
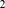
FCs for all five contrasts were calculated and plotted (as listed in Table S3). The dotted line represents the linear regression line. Pearson correlation and the corresponding *p-value* are displayed in the plot.

To verify the microarray results, five genes were randomly chosen among the DE genes (microarray) and analyzed by qRT-PCR. The results showed a high, significant correlation between the 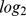 fold changes obtained by qRT-PCR and those obtained by microarray (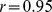, 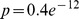: Fig. 7).

### Intestinal microbiota

The intestinal microbiota is intrinsically related to the onset and progress of diseases related to intestinal inflammation. We decided to characterise the microbial communities of individual fish by using PCR-DGGE based on the V6–V8 region of the *16S rRNA* gene followed by sequencing of selected bands. We sequenced 55 different bands, which represented six different species (Dataset S4). In the CV group, three bands were identified as *C.vulgaris* which, although it is an eukariotic organism, possess a copy of the *16S rRNA* gene [40]. Additionally, we identified four bands to represent chloroplasts from soy in all except the FM group. Since all these bands represented artefacts of the diets, they were excluded from subsequent analysis.

**Figure 8:**
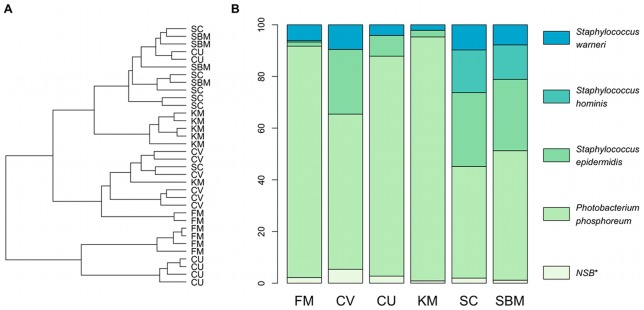
Identification of dominant bacteria in distal intestine. **A:** Dendrogram showing the similarities between 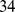 DGGE profiles of PCR-amplified fragments of the 16S rRNA gene (V6–V8 region). The dendrogram was created using the Jaccard similarity coefficient as distance measure and Ward's clustering algorithm. **B:** Relative bacterial abundance in the distal intestine of the six different feeding groups. Abundance for a given bacterial species was calculated using the DGGE band intensities. NSB*  =  non sequenced DGGE bands.

Clustering of banding patterns revealed two main clusters (Fig. 8A). The first cluster mainly contained samples from the groups FM, CU and CV while the second cluster mainly contained samples from the groups KM, SC and SBM. Overall, the results of the clustering showed that the dietary groups, with the exception of SC and SBM, were separate from each other. The groups SC and SBM formed one cluster, which also contained two samples from the CU group. We could observe that the relative abundance of microbiota in the groups was different as a result of the different diets (Fig. 8B). The FM, CV, CU and KM groups were dominated by the phylum *Proteobacteria* (*Photobacterium phosphoreum*), while the phylum *Firmicutes* (*Staphylococcus warneri*, *S.hominis* and *S.epidermidis*) only accounted for a small fraction. In contrast to these groups, both the SC and SBM groups were dominated by *Firmicutes*, while *Proteobacteria* was less abundant.

## Discussion

In the present study, we investigated the potential of four different microbial ingredients (three yeasts and one microalgae) to counteract SBMIE in Atlantic salmon. Our results show that both CV and CU were functional ingredients that were highly effective to counteract SBMIE. KM showed limited efficiency and SC had virtually no effects. The beneficial effects of the microbial ingredients were observed on the level of morphological changes, epithelial cell proliferation in the crypts and global gene expression in the distal intestine. To some extent a dietary effect could also be seen in the abundance of specific intestinal bacteria.

Pathological changes in the distal intestine of fish suffering SBMIE, condition the loss of large amounts of epithelial cells. This effect is likely causative for the lower relative weight of the distal intestinal segment, found in both the SC and SBM groups and which concurs with the results of previous SBMIE experiments [41], [42]. The high, positive correlation that we found between the relative length of PCNA positive cells and the pathology scores indicates a mechanism by which the intestine attempts to compensate for this loss of cells by increasing the proliferation of epithelial cells in the distal intestine. Such a mechanism is further supported by our gene expression data indicating increased cellular turnover in the groups suffering from SBMIE. Increased epithelial cell proliferation has been observed earlier in SBMIE [17]–[19].

We found a significant higher expression of the chemokine signalling pathway and the leucocyte transendothelial migration in the groups suffering SBMIE in our experiment, which may be indicative for recruitment of T-cells that has been previously shown to occur in SBMIE [11], [18]. However, we found relatively few affected immune system related pathways in the gene set enrichment analysis. One reason for this could be that annotations from KEGG are generally biased towards metabolic pathways [23].

In our experiment, we identified several pathways that were linked by previous studies to SBMIE in Atlantic salmon. Those pathways were: amino acid-, fatty acid- and drug metabolism (P450), as well as cell adhesion molecules, lysosome and peroxysomal processes, immune response [10], [43] and bile secretion [29]. All these pathways were strongly regulated in both the SC and SBM group, less strong in the KM group and even further reduced in the CV and CU groups. Pathway regulation in both the CV and CU groups was in most cases indistinguishable from the FM group, thus confirming a healthy intestine.

In addition, we identified new pathways related to SBMIE. We found an up-regulation of the NLR pathway in both the SBM and the SC groups. This observation seems to support the results of a previous study, showing up-regulation of *MyD88* in SBMIE [9], which is activated by Toll-like receptors (TLRs). Both TLRs and NLRs are important receptors recognising microbial components and thereby activating the innate immune response [31], [44]. Increased paracellular permeability of the intestinal epithelium may result from decreased expression of tight junction proteins that we found in the SC and SBM group. Experiments in Atlantic salmon have indeed shown that SBM increases the permeability of the distal intestine [42]. Hence, suggesting that activation of TLRs and NLRs may result from increased amounts of microbial antigens that have breached the barrier.

Previous work using mice deficient of the NLR NOD2 has shown that NOD2 regulates the expression of antimicrobial peptides [45] However, when assessing the expression of antimicrobial peptides in the distal intestine of Atlantic salmon, we found that only two of the seven antimicrobial peptides measured, displayed up-regulation in the SBM and SC group while the remaining five antimicrobial peptides were down-regulated. Reduced expression of antimicrobial peptides, as found in humans suffering IBD, can result in a weakened intestinal barrier function to intestinal microbes and may cause the pathophysiology of the chronic inflammation [46], [47]. In mice, tryptophan metabolism and especially the tryptophan transporter ACE2 are crucial for providing metabolites for the production of antimicrobial peptides as well as regulating their expression [25]. Hence, we suggest that expression of most antimicrobial peptides in the distal intestine of the groups suffering most from SBMIE (SC and SBM) could be governed by the trypthophan metabolism pathway.

In mammals, specialised secretory cells (Paneth cells) are a main source for antimicrobial peptides in the intestine [39]. Although Paneth cells appear to be absent in teleost species, mast cells and rodlet cells (secretory cells in the intestinal epithelium) have been shown to contain antimicrobial peptides and have been proposed to have Paneth cell like functions [48], [49]. SC as well as the SBM group showed dramatically reduced gene expression of the antimicrobial peptides *LYG* and *LECH* (a C-type Lectin). The bactericidal properties of LECH are not well characterised to date, however, Lysozyme is well known to show bactericidal activities, mostly against Gram-positive bacteria [39]. Hence, we speculate that the reduced expression of these antimicrobial peptides in the SC and SBM groups, could be the cause for the higher relative amount of Gram-positive bacteria (*Firmicutes*) that was found in these groups. The increased relative abundance of *Firmicutes* in the inflamed fish intestine is consistent with studies in rainbow trout [12], [50] and Atlantic salmon (Reveco *et al.* unpublished data). It should be mentioned that the contribution of the microbiota to the pathogenesis of SBMIE has been questioned based on one experiment in Atlantic salmon showing that the broad-spectrum antibiotic oxytetracycline could not mitigate the inflammation when administered together with SBM [51]. However, the oxytetracycline treatment only decreased the number of adherent bacteria.

Imbalance in the intestinal microbiota, or dysbiosis, is now a widely accepted contributing factor to the pathogenesis of IBD [16], a condition that shows similarities to SBMIE. Indeed it has been demonstrated in mice that deficiency of the antimicrobial peptides 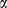-defensin is related to increased abundance of *Firmicutes* in the intestine [52].

It should be noted that this connection between commensal microbiota and histopathology appears to be quite clear in the SC and SBM groups, it was less clear in case of the KM group. A reason for this result could be that the samples for intestinal microbiota originated from individuals where neither histopathology nor expression of antimicrobial peptides were assessed. The KM group showed the largest variation with respect to histopathology, thus it appears possible that that the relative abundance of microbiota in this group could be the results of coincidental sampling of individuals with low histopathology scores.

*M.capsulatus* is another ingredient found to be efficient to counteract SBMIE in Atlantic salmon [17]–[19]. Furthermore, a recent study showed attenuation of DSS-induced colitis in mice as a result of feeding *M.capsulatus* [53] Based on the morphological changes in the intestine, the epithelial cell proliferation in the crypts of the distal intestine and the highly similar effects on gene expression (Skugor *et al.*, unpublished data) it appears that CV and CU are as effective as *M.capsulatus* to counteract SBMIE in Atlantic salmon. This is remarkable, because it shows that ingredients made from phylogenetic distant clades (yeasts, microalgae and bacteria) when fed to Atlantic salmon all show similar efficiency of counteracting SBMIE, whereas ingredients made from phylogenetic close species (such as CV, KM and SC) show large variations in their effects. Based on these similarities, we suggest that there is a common mechanism, underlying the potential of CV, CU and also *M.capsulatus* to counteract SBMIE. In this context, it is interesting to note that feeding *M.capsulatus* in combination with SBM increases expression of the antimicrobial peptides *LYSC2* and the tryptophan transporter *ACE2* (Skugor *et al.*, unpublished data). Furthermore, SBM diet has a low tryptophan digestibility [54] and *M.capsulatus* is particular rich in tryptophan [55].

In conclusion, we found that that inclusion of CU or CV in diets containing SBM counteracts enteropathy in Atlantic salmon. KM partialy reduced enteropathy, while SC did not have any beneficial effects in our experiment. This is supported by an overall high correlation between histopathology scores, immunohistpathology, gene expression and KEGG pathway regulation. Inclusion of CV, CU or KM affected the bacterial composition in the distal intestine, particular by reducing the relative amount of *Firmicutes* bacteria, suggesting that intestinal microbiota are an important factor for the pathophysiology of SBMIE. Given the current results, we propose that the effects of CV and CU are linked to the maintenance of intestinal homeostasis.

## Materials and Methods

### Ethics statement

The feeding trial was carried out at the Nofima research station in Sunndalsø ra (western Norway), which is an approved facility under the Norwegian Animal Research Authority (NARA). Stunning and sampling of fish were performed in accordance with the Norwegian Animal Welfare Act. Fish were treated as production fish up to the point of tissue sampling which was done only after fish were put to death. Hence, no NARA approval was required required according to Dr. G. Bæ verfjord, member of the national NARA board and local NARA officer at Nofima.

### Fish, rearing conditions and sampling

Non-vaccinated post smolt Atlantic salmon (*Salmo salar* L.) were fed either a FM diet (negative control), a SBM diet (positive control) or one of the four microbial ingredients in combination with SBM (Table. 1) for four weeks. The fish were randomly distributed into 18 circular fibreglass tanks at a density of 20 fish per tank. The tanks contained 460 L seawater (10.3°C, NaCl: 32.5 g/L) which was pumped in at 18 L water/min and received 24 h light per day. The six experimental diets were fed continuously by automatic feeders to triplicate tanks. Feed provided was the same for all groups. For feed preparation please refer to Romarheim *et al* [19].

The fish were anesthetized with tricaine methanesulfonate (60 mg/L) and killed by a sharp blow to the head at the termination of the experiment. The body and organ weights for all 20 fish/tank (60 fish/group) were recorded. In addition, individual tissue samples were taken from seven fish/tank (21 fish/group). Tissue samples from the distal intestine of five fish/tank (15 fish/group) were sampled for histology and immunohistochemistry. Four of those five fish from each tank (12 fish/group) were further sampled for RNA. Digesta/feces samples for PCR-DGGE were taken from the distal intestine of two fish/tank (six fish/group). Histology/immunohistology samples were fixed in 10% neutral phosphate buffered formalin and brought to 70% ethanol after 72 h. RNA and PCR-DGGE samples were snap frozen in liquid nitrogen and stored at −80°C.

### Histological examinations

Formalin fixed samples of the distal intestine were embedded in paraffin according to histological standard procedures. The samples were cut using a microtome and the transverse sections were subsequently stained with hematoxylin and eosin. Sections were evaluated by four different criteria:

- ***Lamina propria***: accumulation of leucocytes such as lymphocytes, granulocytes and eosinophilic granule cells in the *lamina propria*.
- **Epithelium**: reduced supra nuclear vacuolisation, reduced cellular height and increased cytoplasmic basophiilia.
- **Atrophy** – reduced height of intestinal folds.
- **Oedema** – accumulation of protein-rich fluid in the *lamina propia*.

Each category was graded on a scale from 0 to 2, where score 0 indicates no sign of abnormal changes, score 1 weak and score 2 severe changes. The sections were coded and scoring was conducted in a blindfolded manner.

### Immunohistochemistry

Paraffin sections were placed on polylysine coated slides (menzel-Glaeser, Germany), and subsequently dried for at least 12 h at 37°C. The dried sections were then deparaffinized in xylene and re-hydrated in a graded ethanol series. Antigene-retrieval was done by autoclaving the sections for 15 min at 121°C in citrate buffer (10 mM/L citric acid monohydrate, pH 6). To prevent unspecified binding of antibodies, the sections were blocked with normal horse serum containing 5% BSA in TBS for 20 min at room temperature. Sections were then incubated with the primary antibody (mouse monoclonal IgG2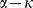 antibody against PCNA (M0879, Dako, Norway) used at a dilution of 1/400 in 1% BSA/TBS buffer) overnight at 4°C. Subsequent incubation with the secondary antibody (biotinylated horse anti Ig (BA 2000, Vector Laboratories, USA), 1/200 in 1% BS/TBS) was conducted 30 min at room temperature. After incubation with the Avidin-HRP complex (PK-4000, Vector Laboratories) in 1% BSA/TBS for 45 min at room temperature, peroxydase activity was detected using the AEC-kit (15 min exposure, Zymed, USA) and sections were mounted. Between all the steps of the procedure the sections were given three washes in PBS. The only exception was after the blocking step, when the sections were not washed but the serum was gently tapped off.

### RNA extraction

Distal intestinal tissue samples (12 samples/group) were homogenized in Quiazol (Qiagen, Germany) using a bead mill (TissueLyser, Qiagen) and total RNA was subsequently extracted and purified using column purification (96 universal Tissue Kit, Qiagen) according to the manufacturer's instructions. Traces of genomic DNA in the samples were eliminated by on-column-DNase digestion (Qiagen). RNA concentrations for all samples were measured for using a NanoDrop 8000 Spectrophotometer (Thermo Scientific, Norway) and RNA quality determined using a Agilent 2100 Bioanalyzer (RNA 6000 NanoLabChip, Agilent, USA). Based on the RIN values, 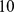 samples per group were selected for subsequent microarray analysis.

### Microarray hybridization

The customized 8×15K oligo (60-mer) Atlantic salmon microarray (SIQ6, Agilent) was used in the experiment. Unless otherwise stated, all equipment and reagents were from Agilent. Amplification and labeling of 100 ng of total RNA was performed using the Low Input Quick Amp Labeling Kit (One-Color). All steps were conducted according to the Agilent protocol (One-Color Microarray-Based Gene Expression Analysis, Version 6.5). Analysis of the microarray images was done in Agilent Feature Extraction (Version 10.7.1.1) Software using the one-color (GE1
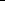
107
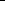
Sep09) protocol.

### Microarray analysis

Normalization and analysis of the data was done in R/Bioconductor [56] using the Bioconductor package “limma” [57]. To generate the data-set, background corrected spot intensity signals (*gProcessedSignal*) were filtered according to the following criteria provided by the Feature Extraction software: *gIsPosAndSignif*, *gIsFeatNonUnifOL*, *gIsWellAboveBG* and *ControlType* (a description of the feature results can be found in the Feature Extraction Software Reference Guide). All control spots were removed from the data set and expression values were normalized by quantile normalization in order to adjust the scale of intensities across arrays [58]. The data was then log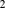 transformed and probe sets showing more than 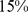 missing values were discarded from the data set, classifying 12065 (80%) probe sets as present. The remaining missing values were imputed using the method based on K Nearest Neighbours [59] implemented in the Bioconductor package “impute”. In total, less than 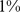 of the values were imputed. Processed and raw microarray data is publicly available at NCBI's GEO repository (Series Nr. **GSE44978**). Differential expression was assessed by fitting the following linear model to each probe set:
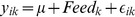
(1)Where 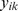 is the 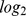 intensity of the 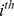 sample of feeding group *k*. 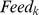 denotes the feeding group and  is the error. Subsequent to fitting the linear model, five contrasts comparing each dietary groups against the FM group were extracted for each probe set. Improved variance estimates were computed using empirical Bayes moderated statistics from the “limma” package [60]. Multiple hypothesis testing was accounted for by using the “nestedF” method implemented in the “limma” package [57]. Probes were considered to be significant for a specific contrast when the adjusted 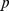-value 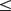
0.05 and the corresponding 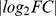 was 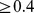.

#### Gene set enrichment (GSE)

KEGG annotations for the probes were obtained through KAAS [61]. Prior to performing GSE analysis, probes mapping to the same gene were collapsed to the probe showing the largest variance (reducing the data set from 3791 to 2631 probes). Subsequently, probes without KEGG annotation were removed from the data set, retaining 1087 genes in 284 different KEGG pathways in the final data set. GSE was analyzed using the GSVA algorithm implemented in the “GSVA” package [62]. Only pathways containing more than 10 and less than 100 genes were considered in the analysis. The resulting GSVA score matrix (KEGG pathway score by sample matrix) was analyzed by fitting the same linear model (Equation 1) to each pathway and extracting the same contrasts as before. The resulting *p-value* (F-statistics) was corrected for multiple testing [63]. KEGG pathways with an adjusted *p-value* 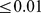 were regarded as significantly regulated (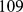 KEGG pathways). *P-values* for the contrasts were corrected for multiple testing [63] simultaneously across all pathways and contrasts. The R Code to reproduce the microarray analysis can be found at: http://www.umb.no/iha/artikkel/specific-microbial-ingredients.

### qRT-PCR

Single strand cDNA was reverse transcribed from total, DNase treated, RNA using oligo-dT primers and the Taq Man reverse transcription Kit (Applied Biosystems, USA). qRT-PCR was performed in 96-well optical plates on a Light-Cycler 480 (Roche, Switzerland). For the PCR reaction 2x SYBR green I master Mix (Roche), 0.41 nM of each primer and the cDNA template were mixed in a total reaction volume of 10 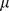
l. Primer sequences are listed in Table S2. A three step PCR protocol with 45 cycles [95°C 15 s; 60°C 15 s; 72°C 15 s] was used. To verify specific amplification a melting curve analysis step was done at the end of the program. All samples were analyzed in duplicates and for each measured gene a standard curve was produced using a serial dilution from a pool of all cDNA samples.

The expression levels were normalised to the expression index of the housekeeping genes *Elongation factor 1* 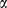 (*EF1* 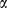) and *Glyceralaldehyde-3-phosphatase* (*GAPDH*), which were found to be stably transcribed in all samples according to the software geNorm [64]. Significance was tested by fitting a ANOVA model (Equation 1) to the data. However, this time 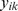 denotes the 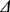
CP value (multiplied by 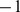 to be interpretable as 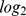 expression value) of the 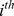 sample of feeding group *k*.

### DNA extraction

Total genomic DNA was extracted from digesta samples of two individuals per tank (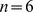 per dietary group) by using the QIAamp DNA stool Mini Kit (Qiagen) following the protocol for isolation of DNA from stool for pathogen detection. The lysis temperature was 95°C to ensure the lysis of Gram-positive bacteria. DNA concentration was determined using a NanoDrop 8000 Spectrophotometer (Thermo Scientific).

### PCR-DGGE

PCR amplification of V6–V8 region of the 16S rRNA genes was conducted using the universal bacterial primers F-968 including a 40-bp GC clamp at the 5'end (F-968GC) and R-1401 [65] on total genomic DNA samples. The PCR reaction mixture used was previously described by Yu and Morrison [65]. After an initial cycle [94°C 4 min; 58°C 1.5 min; 72°C 1.5 min], a touchdown-PCR was conducted as follows: [94°C 30 s; 58°C 30 s; 72°C 1 min] followed by 5 cycles with 1°C/cycle decrement. Then, 28 cycles of [94°C 45 s; 53°C 45 s; 72°C 1 min] and a final extension at 72°C 10 min. Successful amplification was confirmed by agarose gel electrophoresis.

DGGE analysis was performed using the INGENYphorU system (Ingeny International BV, Netherlands). PCR products were resolved on 8% polyacrylamide gels (37.5:1) in 1X Tris-acetate-EDTA (TAE) buffer (40 mM Tris-acetate, 20 mM acetic acid, 1 mM EDTA) with a gradient of 42–58% of urea and formamide (100% denaturant corresponding to 7 M urea and 40% [v/v] formamide). DGGE gels were run at 60°C with a constant voltage of 75 V (1200V-h). The DGGE gels were stained with SYBRGold Nucleic Acid Gel Stain (Invitrogen Dynal AS, Oslo, Norway) in 1X TAE buffer for 10 min and scanned on the Gel Doc XR System (Bio-Rad laboratories, Norway). After normalization of the gels DGGE profiles were documented (as area under the Gaussian curve approximating for each band) using GelCompar II V6.0 software (Applied Maths Inc., USA). Selected DGGE bands were excised and the DNA eluted in water. DNA was re-amplified using F-968 and R-1401 primers and subsequently purified using a MultiScreen PCR
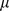
96 Filter Plate (Millipore A/S, Norway). The re-amplified fragments were then sequenced using BigDye Terminator v3.1 Cycle Sequencing Kit on an ABI 3730 DNA analyzer (Applied Biosystems, Foster, USA) with the F-968 and R-1401 primers. The sequences were edited and aligned by using BioEdit and ClustalW, respectively. The resulting sequences (359–401 bp) were compared and identified by using BLASTN searches in the GenBank based on the nearest known relative. The sequences are available at the European Nucleotide Archive (http://www.ebi.ac.uk/ena/); accessions: HG326234–HG326252).

### Data analysis

The *non-parametric* scores from the histological examinations were analyzed using the Kruskal-Wallis one-way ANOVA by ranks and the *non-parametric* Nemenyi-Dunn test [66] as *post-hoc* test. Morphometry was done on PCNA stained sections. The length of the stretches of PCNA positive epithelial cells, located in the crypts of the intestinal folds, were measured using ImageJ software [67] and expressed as:
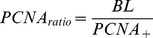
(2)Where 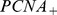 is the length of the stretches of PCNA positive cells and 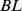 is the base line, the total length of the intestinal folds. In order to asses differences between the groups, a one-way ANOVA test was conducted on 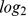 transformed PCNA ratios, followed by a Tukey HSD test as *post-hoc* test. Production data was analyzed by one-way ANOVA followed by a Tukey HSD test as *post-hoc* test. Relative bacterial abundance was determined by first transforming the band intensities (area under the Gaussian curve approximating a DGGE band) in each individual sample (lane) to percent, followed by averaging the band intensities (in %) for each bacterial species, across all samples from the same dietary group.

All statistical tests were conducted within the statistical software R (Version 2.15.1, http://cran.r-project.org/).

## Acknowledgments

The authors would like to thank Frank Sundby and Kyla Randall for their support during collection and processing of the fish samples, Inger Rudshaug for excellent work with histology/immunohistochemistry and Dr. Aleksei Krasnov for providing the microarray. Furthermore, we would like to thank You Song and Dr. Knut Erik Tollefsen for providing technical help during the scanning.

## Supporting Information

Figure S1Histology of the distal intestine of Atlantic salmon fed diets with FM (fish meal), CV (*Chlorella vulgaris*), CU (*Candida utilis*), KM (*Kluyveromyces marxianus*), SC (*Saccharomyces cerevisiae*) or SBM (soybean meal). As compared with the FM (control) SBM shows an accumulation of leukocytes (arrows) in the tissue between the epithelium and the stratum compactum (*), a reduced vacuolization and cellular height of the epithelial cells and increased cytoplasmic basophilia of the epithelial cells (filled arrowheads), atrophy with shortening of the intestinal folds, all changes corresponding to SBM induced enteropathy (SBMIE). Open arrowheads indicate a normal, vacuolated epithelium. CV and CU images shown here are considered to be i n the normal range. KM and SC show changes typical for SBMIE. The sections were stained with hematoxylin and eosin. Magnification is the same for all images; scale bar in the upper left corner is 200 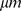.(TIF)

Figure S2 Immunohistochemistry for PCNA (proliferating cell nuclear antigen) of the distal intestine of Atlantic salmon fed diets with FM (fish meal), CV (Chlorella vulgaris), CU (Candida utilis), KM (Kluyveromyces marxianus), SC (Saccharomyces cerevisiae) or SBM (soybean meal). As compared with the FM, CV and CU the diets KM, SC, and SBM show an increase in the size of the population staining for PCNA (brown). Magnification is the same for all images; the scale bar in the upper left corner is 200 micrometers.
(TIF)

Dataset S1
**Differentially expressed probes**, identified by comparing expression levels of the study groups (SBM: soybean meal; CV: *Chlorella vulgaris*; CU: *Candida utilis*; KM: *Kluyveromyces marxianus*; SC: *Saccharomyces cerevisiae*) against those of the negative control group (FM: fish meal). ID (Col. A)  =  probe ID; CV.FM – SBM.FM (Col. B–F)  =  log
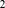
FCs of the respective dietary group relative to the FM group; AveExp, F, P.Value, adj.P.Val (Col. G–J)  =  Average log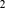 signal; F-value, P-value, adjusted P-value; CV-FM – SBM-FM (Col. B-F)  =  boolean indicator (1 =  probe DE for the respective contrast, 0 =  not DE).(XLS)

Dataset S2 **Gene set enrichment analysis results** for all KEGG pathways with more than 10 and less than 100 genes. CV-FM – SBM-FM (Col. A–E)  =  Average pathway enrichment score of the respective dietary group relative to the FM group; KEGG pathway (Col F)  =  KEGG Orthology (KO) pathway ID; Name (Col G)  =  KEGG Orthology (KO) pathway Name; L1–L2 (Col H-J)  =  KEGG pathway meta information; F, P.Value, adj.P.Val (Col. J–L)  =  F-value, P-value, adjusted P-value; Signif:CV-FM – Signif:SBM-FM (Col M-Q) =  boolean indicator (1 =  pathway DE for the respective contrast, 0 =  not DE)
(XLS)

Dataset S3
**Gene set enrichment analysis results II**, showing genes and their differential expression per KEGG pathway. probe ID (Col. A)  =  probe ID; kegg_orthology (Col. B)  =  KEGG orthology annotation for the gene; KEGG_pathway (Col. C)  =  KEGG Orthology (KO) pathway ID; gene_name (Col. D)  =  Gene name; gene_symbol (Col. E)  =  Gene symbol; CV-FM – SBM-FM (Col. F–J)  =  log
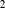
FCs of the gene (of the respective dietary group) relative to the FM group; AveExp, F, P.Value, adj.P.Val (Col. K–N)  =  Average log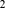 signal; F-value, P-value, adjusted P-value(XLS)

Dataset S4 **Blast results for sequenced DGGE bands.**
(XLS)

Table S1
**Correlation matrix** showing the spearman correlation of the four different histological scores (*Lamina propria*, Epithelium, Atrophy, Oedema) and the 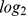 transformed length of PCNA positive regions in the crypts of the distal intestine.(PDF)

Table S2 **qRT-PCR primer**
(PDF)

Table S3 **Microarray validation**
(PDF)
