# Supplementary figures and images for: Candida utilis and Chlorella vulgaris Counteract Intestinal Inflammation in Atlantic Salmon (Salmo salar L.)
Source: PLoS One. 2013 Dec 27;8(12):e83213. doi: 10.1371/journal.pone.0083213 (PMC3873917; doi:10.1371/journal.pone.0083213)

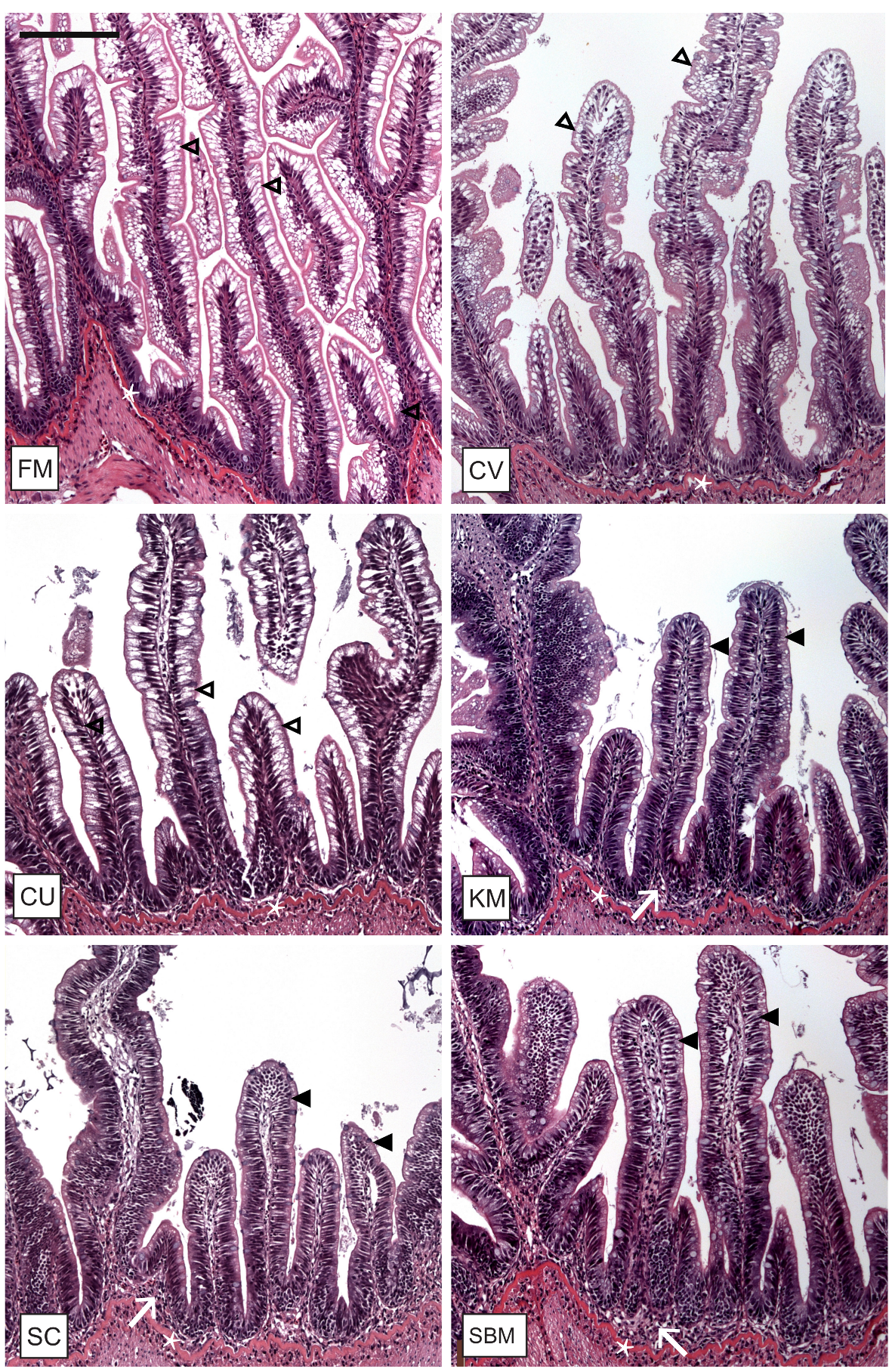

Supplement: Figure S1 — Histology of the distal intestine of Atlantic salmon fed diets with FM (fish meal), CV (Chlorella vulgaris), CU (Candida utilis), KM (Kluyveromyces marxianus), SC (Saccharomyces cerevisiae) or SBM (soybean meal). As compared with the FM (control) SBM shows an accumulation of leukocytes (arrows) in the tissue between the epithelium and the stratum compactum (*), a reduced vacuolization and cellular height of the epithelial cells and increased cytoplasmic basophilia of the epithelial cells (filled arrowheads), atrophy with shortening of the intestinal folds, all changes corresponding to SBM induced enteropathy (SBMIE). Open arrowheads indicate a normal, vacuolated epithelium. CV and CU images shown here are considered to be i n the normal range. KM and SC show changes typical for SBMIE. The sections were stained with hematoxylin and eosin. Magnification is the same for all images; scale bar in the upper left corner is 200 . (TIF) [file pone.0083213.s001.tif]

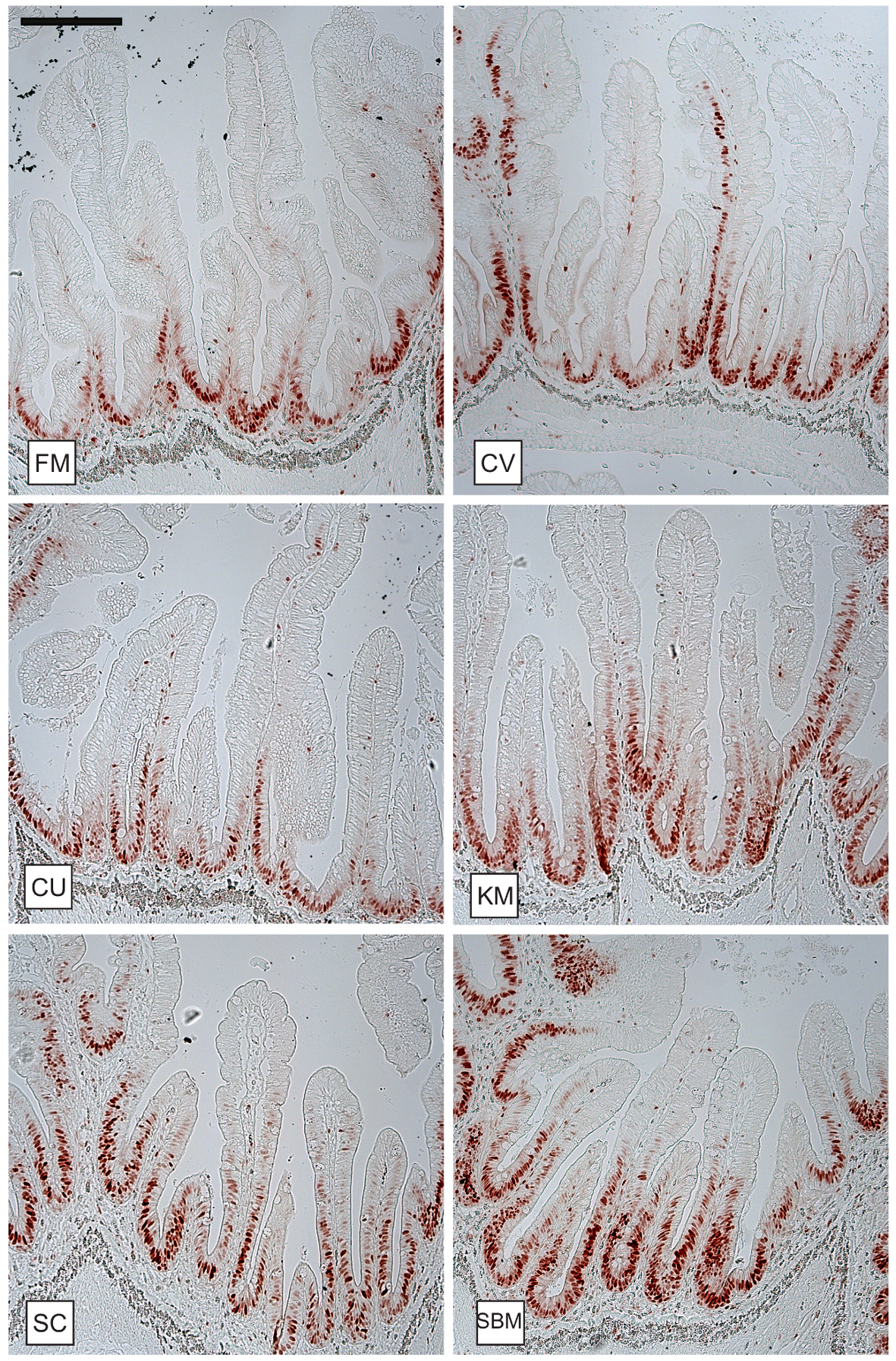

Supplement: Figure S2 — Immunohistochemistry for PCNA (proliferating cell nuclear antigen) of the distal intestine of Atlantic salmon fed diets with FM (fish meal), CV (Chlorella vulgaris), CU (Candida utilis), KM (Kluyveromyces marxianus), SC (Saccharomyces cerevisiae) or SBM (soybean meal). As compared with the FM, CV and CU the diets KM, SC, and SBM show an increase in the size of the population staining for PCNA (brown). Magnification is the same for all images; the scale bar in the upper left corner is 200 micrometers. (TIF) [file pone.0083213.s002.tif]
