# Supplementary material for: Candida utilis and Chlorella vulgaris Counteract Intestinal Inflammation in Atlantic Salmon (Salmo salar L.)
Source: PLoS One. 2013 Dec 27;8(12):e83213. doi: 10.1371/journal.pone.0083213 (PMC3873917; doi:10.1371/journal.pone.0083213)
Supplement: Table S1 — Correlation matrix showing the spearman correlation of the four different histological scores (Lamina propria, Epithelium, Atrophy, Oedema) and the transformed length of PCNA positive regions in the crypts of the distal intestine. (PDF) [file pone.0083213.s007.pdf]

	L.prop	Epit.	Atrophy	Oedema
Epit.	0.94			
Atrophy	0.92	0.95		
Oedema	0.81	0.87	0.87	
$\log_2$(PCNA)	0.68	0.70	0.67	0.61
