# Supplementary material for: Candida utilis and Chlorella vulgaris Counteract Intestinal Inflammation in Atlantic Salmon (Salmo salar L.)
Source: PLoS One. 2013 Dec 27;8(12):e83213. doi: 10.1371/journal.pone.0083213 (PMC3873917; doi:10.1371/journal.pone.0083213)
Supplement: Table S2 — qRT-PCR primer (PDF) [file pone.0083213.s008.pdf]

Table S2.

Acc	Gene Name	Forward 5’-3’	Reverse 5’-3’
BT050045	Glyceraldehyde 3-phosphate dehydrogenase	AAGTGAAGCAGGAGGGTGGAA	CAGCCTCACCCCATTTGATG
BG933853	Elongation factor 1-alpha	TGCCCCTCCAGGATGTCTAC	CACGGCCCACAGGTACTG
CB514404	Lysosomal alpha-mannosidase	ACTGCAGCCTCTCATCTCGT	TGCCTGTAGCCCAGAGAACT
CK885977	Keratin, type I cytoskeletal 18	ATGGGCAATGAGAAGTTTGC	TCCAGGTTCCTCACCATCTC
DY692675	Hepcidin-1	AGTCATGGATATGGCGAAGG	GTGCGATGTTGATGGTGATG
EG845759	Phosphatidylinositol 4-phosphate 1 5-kinase-like protein	ATTCCATGGCCAGAGTGTTC	TAAAGCCCATGGACCAAGAC
TC113038	Sialin	CACCAACACTTTCGCGACTA	TGCCAAACAAGGTGAAGACA
AY360357	cathelicidin 2	GGAGACGCTCTGCAGTAAGG	AGCATCTTCTGCCTCCATGT
EG781611	Defensin b4	CCATTTGGACCTTTTGGATG	TGGAGGAACATCAATGCAGA
