# Supplementary material for: Candida utilis and Chlorella vulgaris Counteract Intestinal Inflammation in Atlantic Salmon (Salmo salar L.)
Source: PLoS One. 2013 Dec 27;8(12):e83213. doi: 10.1371/journal.pone.0083213 (PMC3873917; doi:10.1371/journal.pone.0083213)
Supplement: Table S3 — Microarray validation (PDF) [file pone.0083213.s009.pdf]

Contrast	log_2FC Microarray	log_2FC qRT-PCR	Gene Name
CV-FM	-0.52	-1.07	Lysosomal alpha-mannosidase
CV-FM	-0.67	-1.36	Sialin
CV-FM	0.17	0.11	Solute carrier family 13 member 2
CV-FM	0.28	0.29	Keratin, type I cytoskeletal 18
CV-FM	0.50	1.14	Phosphatidylinositol 4-phosphate 5-kinase-like protein 1
CU-FM	-0.86	-1.08	Lysosomal alpha-mannosidase
CU-FM	-0.58	-0.88	Sialin
CU-FM	-0.66	-0.47	Solute carrier family 13 member 2
CU-FM	0.48	0.11	Keratin, type I cytoskeletal 18
CU-FM	0.57	0.63	Phosphatidylinositol 4-phosphate 5-kinase-like protein 1
KM-FM	-0.74	-0.77	Lysosomal alpha-mannosidase
KM-FM	-0.97	-1.10	Sialin
KM-FM	-0.05	0.24	Solute carrier family 13 member 2
KM-FM	0.49	0.68	Keratin, type I cytoskeletal 18
KM-FM	0.49	1.06	Phosphatidylinositol 4-phosphate 5-kinase-like protein 1
SC-FM	-2.16	-2.36	Lysosomal alpha-mannosidase
SC-FM	-0.80	-1.29	Sialin
SC-FM	-2.70	-2.13	Solute carrier family 13 member 2
SC-FM	0.79	0.91	Keratin, type I cytoskeletal 18
SC-FM	1.08	1.51	Phosphatidylinositol 4-phosphate 5-kinase-like protein 1
SBM-FM	-2.15	-2.28	Lysosomal alpha-mannosidase
SBM-FM	-1.07	-0.45	Sialin
SBM-FM	-2.09	-2.25	Solute carrier family 13 member 2
SBM-FM	1.05	0.80	Keratin, type I cytoskeletal 18
SBM-FM	1.06	0.77	Phosphatidylinositol 4-phosphate 5-kinase-like protein 1
